# From Waste to Taste: Dynamic Interaction of Grape Stems with Wine Off-Odors

**DOI:** 10.3390/foods15101707

**Published:** 2026-05-13

**Authors:** Giovanni Luzzini, Jessica Anahi Samaniego Solis, Jacopo Nicola Bergamo, Naíssa Prévide Bernardo, Davide Slaghenaufi

**Affiliations:** 1 Department of Biotechnology, University of Verona, Villa Lebrecht, Via Della Pieve 70, 37029 San Pietro in Cariano, Italy; 2 Department of Business Management and Marketing, University of Vigo, Calle Benito Corbal 45, 36001 Pontevedra, Spain; 3 Grape and Wine Technological Centre, Experimental Farm of Caldas, Agricultural Research Company of Minas Gerais, Avenida Santa Cruz, 500, Caldas 37780-000, Brazil

**Keywords:** stems, withering, C_6_ alcohols, pyrazine, methanethiol, circular economy, tainted odors

## Abstract

Within the circular economy framework, grape stems, a major winemaking by-product, are increasingly recognized for their potential to modulate wine composition despite some criticalities. This study aimed to investigate fresh and withered stems as both sources of compounds and adsorbents of off-odors. Corvina and Cabernet Sauvignon stems were tested under three conditions: fresh, and 20% and 40% weight loss. Over 14 days of maceration in red wine, the release kinetics of key enological parameters, including pH, ethanol, total phenolics, methoxypyrazines, and C_6_ alcohols, were investigated. Concurrently, the adsorption capacity for methanethiol was evaluated. Results indicated that stems significantly influence wine composition by increasing pH and phenolic content while reducing ethanol, with variability associated with the withering treatment. Withered stems showed reduced release of herbaceous pyrazines compared to fresh stems. Stems demonstrated a high affinity for methanethiol, resulting in a significant decrease greater than that observed with commercial enological tannins, known for their ability to reduce reductive mercaptans. This decrease was primarily driven by direct adsorption onto the solid stem matrix, with a secondary contribution from leached soluble compounds. This work provides new insights into the chemical interplay between grape stems and wine, highlighting their valorization potential as a sustainable tool to manage wine composition and mitigate sensory defects.

## 1. Introduction

In traditional winemaking, grape stems are typically removed before fermentation to prevent the extraction of compounds that can negatively affect wine quality [1,2,3]. Despite this long-standing practice, research has increasingly focused on the controlled use of stems, suggesting they could serve as a tool to improve wine complexity and stability [3,4]. While their structural composition is primarily based on water, cellulose, hemicellulose, lignin, and proteins, their enological relevance arises from the high content of polymeric phenols and flavanol monomers they contain [5,6,7,8,9]. The increase in tannin concentration, which heightens astringency, resulting from their interaction and precipitation with salivary proteins, and bitterness, is the principal reason stems have been historically excluded from the winemaking process [10]. However, this characteristic can be advantageous for certain grape varieties that are inherently deficient in tannins and polyphenols. In such cases, the introduction of stems can contribute positively to the wine’s features, increase its antioxidant capacity, enhance its sensory profile, and improve its longevity through color stabilization via slow polymerization reactions with anthocyanins [7,11].

Another significant concern that has discouraged the use of whole clusters is the potential development of herbaceous or vegetal off-odors [12]. This is attributed to the release of specific volatile organic compounds from the stems, most notably C_6_ alcohols and various pyrazines [4,13]. Methoxypyrazines, particularly IBMP, are characteristic of Cabernet Sauvignon and often impart a distinct green bell pepper aroma. However, at high concentrations, they are considered a sensory defect due to their overwhelming vegetative character (Roujou de Boubée et al., 2000 [14]). C_6_ alcohols are generally considered unpleasant or defective because of their pungent, ‘cut grass’, and vegetal character, masking the fruity notes of the wine [15].

Beyond their direct impact on phenolic content and aroma, stems could exert complex influences on fermentation kinetics and the final chemical composition of the wine. Comparative analyses of fermented must with and without stems have shown that their presence can accelerate fermentation, resulting in wines with lower residual sugar levels. This phenomenon is hypothesized to be linked to the physical structure of the stems, which may enhance oxygen transfer and thereby stimulate yeast metabolism [16]. Furthermore, stems can act as a thermal buffer, mitigating temperature extremes within the fermenting mass and consequently lowering the risk of stuck or sluggish fermentations [16]. Their inclusion also has measurable effects on fundamental enological parameters, including pH and titratable acidity [17,18], ethanol concentration [7,19], color intensity and stability [7], and overall taste perception [19,20]. More recently, the potential antimicrobial properties of stems have been investigated to reduce or partially replace sulfur dioxide (SO_2_) additions during pre-fermentative stages [21].

Wang et al., 2025 [22,23,24,25,26,27,28], have performed important research. Concurrently with scientific progress, the food industry has shifted towards sustainability, especially since the 2015 Sustainable Development Goals. The wine sector, known for its large environmental footprint [22], is restructuring to align with circular economy principles, which aim to boost productivity while minimizing waste [23]. This model departs from the linear “take-make-dispose” system, advocating for regeneration and, in broader views, social equity [24,25,26,27]. Central to this are the “3Rs”: Reduce, Reuse, Recycle. In the wine industry, organic by-products such as grape pomace—the solid residue from pressing (skins, pulp, seeds, stems)—are seen as valuable resources [28]. Current pomace uses include biogas, functional flours, cosmetics ingredients, and organic fertilizers [23]. Unlike energy-intensive methods, this study characterizes the properties of stems to find low-impact, circular-economy-aligned uses for them.

Grape withering is a traditional process employed in the production of passito-style wines, most notably in the Valpolicella region of northern Italy, near Verona [29]. This post-harvest dehydration process significantly alters the composition of the grape cluster. As the berries lose water, the stems also dehydrate, leading to increased concentrations of polyphenols and organic acids, along with metabolic transformations in their volatile organic compound profiles [30,31].

While the enological effects of fresh stems have been the subject of extensive research [7,12,21,32,33,34], limited information is available regarding withered stems. Specifically, there is a lack of data concerning the release or interaction kinetics of key chemical parameters (e.g., pH, ethanol, polyphenol content) and major off-odors, such as pyrazines and methanethiol [35]. Preliminary findings from a prior study indicated that withered stems could significantly influence on certain varietal aroma compounds [36].

The aim of this study was to evaluate the capacity of grape stems of different genetic origins (Corvina and Cabernet Sauvignon), subjected to different withering treatments (fresh, 20%, and 40% weight loss), to release or adsorb sensory-active compounds related to wine defects, such as pyrazines and C_6_ alcohols for herbaceous off-odors, and methanethiol for reductive off-odors.

Unlike previous studies, which have mainly focused on fresh stems, this work introduces significant novelty by comparing the effects of withering on the dual-release/adsorption capacity of stems. The relevance of this study lies in providing winemakers with a sustainable, circular economy-based strategy to mitigate sensory defects (herbaceous and reductive) without chemical interventions, while valorizing a major winemaking by-product.

## 2. Materials and Methods

### 2.1. Sample Preparation

#### 2.1.1. Evaluation of Adsorption and Release Kinetics by Grape Stems

For this study, Corvina (**CA**) and Cabernet Sauvignon (**CS**) grape stems were employed and subjected to three different treatments. Fresh (**FR**), withered in a ‘*fruttaio*’ until a 20% of weight loss was achieved (**20**), and withered until a 40% of weight loss was achieved (**40**). The *fruttaio* is a traditional Valpolicella drying room where grapes are dehydrated prior to their use in crafting Valpolicella PDO wines.

Fifteen grams of fresh stems, or a weight of withered stems equivalent to 15 g of fresh stems, were added to 100 mL of red wine (pH 3.48, total acidity 6.7 g/L, ethanol 10.7%, SO_2_ 25.4 mg/L) supplemented with 60 µg/L of methanethiol (MeSH). Additionally, a control was prepared without any stems added. The samples were prepared in triplicate and analyzed after 1, 3, 7, and 14 days of storage at 20 ± 1 °C. Samples and their humidity are reported in Table 1.

#### 2.1.2. Evaluation of the Adsorption Capacity of Grape Stems

In this study, the capacity of grape stems to absorb volatile compounds was investigated. Specifically, 150 g of fresh Corvina grape stems (stalks) were added to a model solution (6.5 g/L of tartaric acid, 10.5% EtOH, pH 3.5, SO_2_ 25 mg/L). After a 14-day storage period at 20 ± 1 °C, the stems were removed from the initial model wine and transferred into a fresh model wine solution. This procedure allowed for the preparation of three distinct sample types: Stem-infused solution: A solution macerated with grape stems for 14 days (**SI**). Exhausted stem solution: A fresh model wine solution to which the previously used (spent) stems were added (**ES**). Stem extract (Stems removed): The liquid fraction obtained after the 14-day maceration was filtered to remove all solid stem residues (**SE**). Additionally, a control sample consisting solely of the model wine solution was prepared. All experimental groups were supplemented with 60 µg/L of MeSH to monitor its concentration over time. The addition of 60 µg/L of MeSH was chosen based on literature evidence, which indicates that this concentration is a realistic and potentially occurring level in wines affected by pronounced reductive off-odors [37,38].

#### 2.1.3. Evaluation of the MeSH Adsorption Capacity of Commercial Enological Tannins

Three different concentrations (25 mg/L, 250 mg/L, and 500 mg/L) of three different enological tannins (T1, T2, and T3) were added to red wine (pH 3.48, total acidity 6.7 g/L, Ethanol 10.7%, SO_2_ 25.5 mg/L) supplemented with 60 µg/L MeSH. Additionally, a control was prepared without the addition of any enological tannins. The samples were prepared in triplicate and, after 1, 3, 7, and 14 days of storage at 20 ± 1 °C, were analyzed. Sample, their botanical origins and concentrations are reported in Table 2.

### 2.2. SPME-GC-MS Analysis of Methanethiol

Methanethiol was analyzed by SPME-GC-MS as described by Slaghenaufi et al. (2021) [39]. Retention indices, quantification ions, limit of detection (LOD), limit of quantification (LOQ), and repeatability of the analyzed compounds are reported in Supplementary Table S1. In order to prevent compound volatilization, wine samples were kept at 4 °C for 24 h prior to analysis. Samples were prepared by adding 100 μL of DMS-d6 internal standard (2 mg/L in ethanol) to 10 mL of wine placed in a 20 mL glass vial containing 3 g of NaCl. Samples were then kept at 4 °C until SPME extraction. Prior to SPME extraction, samples were equilibrated for 1 min at 40 °C, then a polydimethylsiloxane-divinylbenzene fiber (PDMS/DVB) (Supelco, Bellafonte, PA, USA) was exposed to the sample headspace for 30 min. Volatile sulfur compounds (VSCs) were desorbed in the injector port at 270 °C for 2 min in splitless mode. GC-MS analyses were performed as reported previously. GC-MS analysis was carried out on an HP 7890A (Agilent Technologies, Santa Clara, CA, USA) gas chromatograph coupled to a 5977B quadrupole mass spectrometer, equipped with a MPS3 autosampler (Gerstel, Müllheim/Ruhr, Germany). Separation was performed using a DB-WAX UI capillary column (30 m × 0.25, 0.25 μm film thickness, (Agilent Technologies, Santa Clara, CA, USA) and helium (6.0 grade) as carrier gas at a constant flow rate of 1.2 mL/min. The GC oven was programmed as follows: started at 35 °C for 5 min, increased to 90 °C at 5 °C/min, and then to 260 °C at 15 °C/min maintained for 2 min. The mass spectrometer was equipped with an electron impact ionization source (EI) (70 eV). The transfer line, the source, and the quadrupole temperature were set to 200 °C, 230 °C, and 150 °C. Mass spectra were acquired in SIM mode. Samples were analyzed in random order. A calibration curve was prepared using seven concentration points and three replicate solutions per point in white wines. 100 µL of DMS-d6 (2 mg/L in ethanol) was added to each calibration solution, which was then submitted to SPME extraction and GC-MS analysis as described for the samples. Calibration curves were obtained using Chemstation software Version C.01.10 (Agilent Technologies, Santa Clara, CA, USA.) by linear regression, plotting the response ratio (analyte peak area divided by internal standard peak area) against concentration ratio (added analyte concentration divided by internal standard concentration).

### 2.3. SPME-GC-MS Analysis of Pyrazine

For quantification of methoxypyrazines, an SPME extraction followed by GC-MS analysis was used, adapting the procedure described by Belancic & Agosin (2007) [40] and Plank et al. (2019) [41]. An amount of 3 g of NaCl, 2.5 mL of water, and 500 µL of a NaOH 2N solution was added to a glass vial, then 7.5 mL of wine and 250 µg/L of the internal standard 4-methoxy-alpha-toluene-thiol (30 µg/L in ethanol) prior to GC-MS analysis. Samples were equilibrated for 5.50 min at 30 °C. Subsequently, SPME extraction was performed using a 65 µm polydimethylsiloxane-divinylbenzene (PDMS/DVB) fiber (Supelco, Bellafonte, PA, USA) exposed to the sample headspace for 45 min. GC-MS analysis was carried out on an HP 7890B (Agilent Technologies, Santa Clara, CA, USA) gas chromatograph coupled to a 5977B quadrupole mass spectrometer, equipped with a MPS autosampler (Gerstel, Müllheim/Ruhr, Germany). Separation was performed using a HP-5ms UI capillary column (30 m × 0.25 mm, 0.25 µm film thickness, Agilent Technologies, Santa Clara, CA, USA) and helium (6.0 grade) as carrier gas at 1.5 mL/min constant flow rate. GC injector at 260 °C and the oven was programmed as follows: at 70 °C for 3 min, raised to 115 °C at 3 °C/min, to 120 °C at 1 °C/min, to 230 °C at 10 °C/min, and then to 250 °C at 20 °C/min and maintained for 20 min. The mass spectrometer was operated in electron ionization (EI) at 70 eV with an ion source temperature of 250 °C and a quadrupole temperature of 150 °C. The mass spectra were acquired in the SIM mode and the calibration curves were prepared for the 3-isopropyl-2-methoxypyrazine (IPMP), 3-secbutyl-2-methoxypyrazine (SBMP), and 3-isobutyl-2-methoxypyrazine (IBMP) pyrazine standards and obtained using Chemstation software (Agilent Technologies, Inc.) by linear regression, plotting the response ratio (analyte peak area divided by internal standard peak area) against concentration ratio (added analyte concentration divided by internal standard concentration).

### 2.4. SPME-GC-MS Analysis of C_6_ Compounds

C_6_ compounds were analyzed using SPME extraction coupled with GC-MS analysis as described by Slaghenaufi et al. (2022) [42]. Five milliliters of wine were placed into a 20 mL glass vial together with 5 mL of water, 3 g of NaCl, and 5 µL of internal standard 2-octanol (4.2 mg/L in ethanol). Samples were equilibrated for 1 min at 40 °C, and then SPME extraction was performed by exposing for 60 min a 50/30 μm divinylbenzene–carboxen–polydimethylsiloxane (DVB/CAR/PDMS) fiber (Supelco, Bellafonte, PA, USA) into sample headspace. Injection was performed in splitless mode by desorbing SPME fiber into the injection port of an HP 7890A (Agilent Technologies, Santa Clara, CA, USA) gas chromatograph coupled to a 5977B quadrupole mass spectrometer, equipped with a MPS3 auto sampler (Gerstel, Müllheim/Ruhr, Germany). Separation was performed using a DB-WAX UI capillary column (30 m × 0.25, 0.25 μm film thickness, Agilent Technologies, Santa Clara, CA, USA). Helium (6.0 grade) was used as a carrier gas at a constant flow rate of 1.2 mL/min. GC oven temperature was initially set at 40 °C for 3 min, then raised to 230 °C at 4 °C/min and maintained for 20 min. The mass spectrometer was operated in electron ionization (EI) at 70 eV with an ion source temperature of 230 °C and a quadrupole temperature of 150 °C. Mass spectra were acquired in synchronous Scan (*m*/*z* 40–200) and SIM mode. Samples were analyzed in random order.

A calibration curve was prepared similarly to the samples using seven concentration points and three replicate solutions per point in model wine with the exception of hexanal and 2-hexanal, which were quantified with a hexanol calibration curve. Calibration curves were obtained by linear regression, plotting the response ratio (analyte peak area divided by internal standard peak area) against concentration ratio (added analyte concentration divided by internal standard concentration).

### 2.5. Enological Parameters Analysis

Folin–Ciocalteu reagent was used to quantify the total phenolics, according to the procedure described by Singleton and Rossi (1965) [43]. The pH was evaluated with a Basic 20+ pH meter (Crison, Barcelona, Spain). Ethanol was quantified with a Lyza 5000 (Anton Paar, Graz, Austria).

### 2.6. Statistical Analyses

ANOVA analysis with post hoc Tukey test (α = 0.05) has been performed using XLSTAT 2023 (Addinsoft SARL, Paris, France). Prior to ANOVA analysis normality test was assessed using the Shapiro–Wilk test, and homogeneity of variances was checked using Levene’s test. All the graphics have been created using Excel 365 (Microsoft, Redmond, WA, USA).

## 3. Results and Discussion

### 3.1. Adsorption and Release Kinetics by Grape Stems

**Figure 1:**
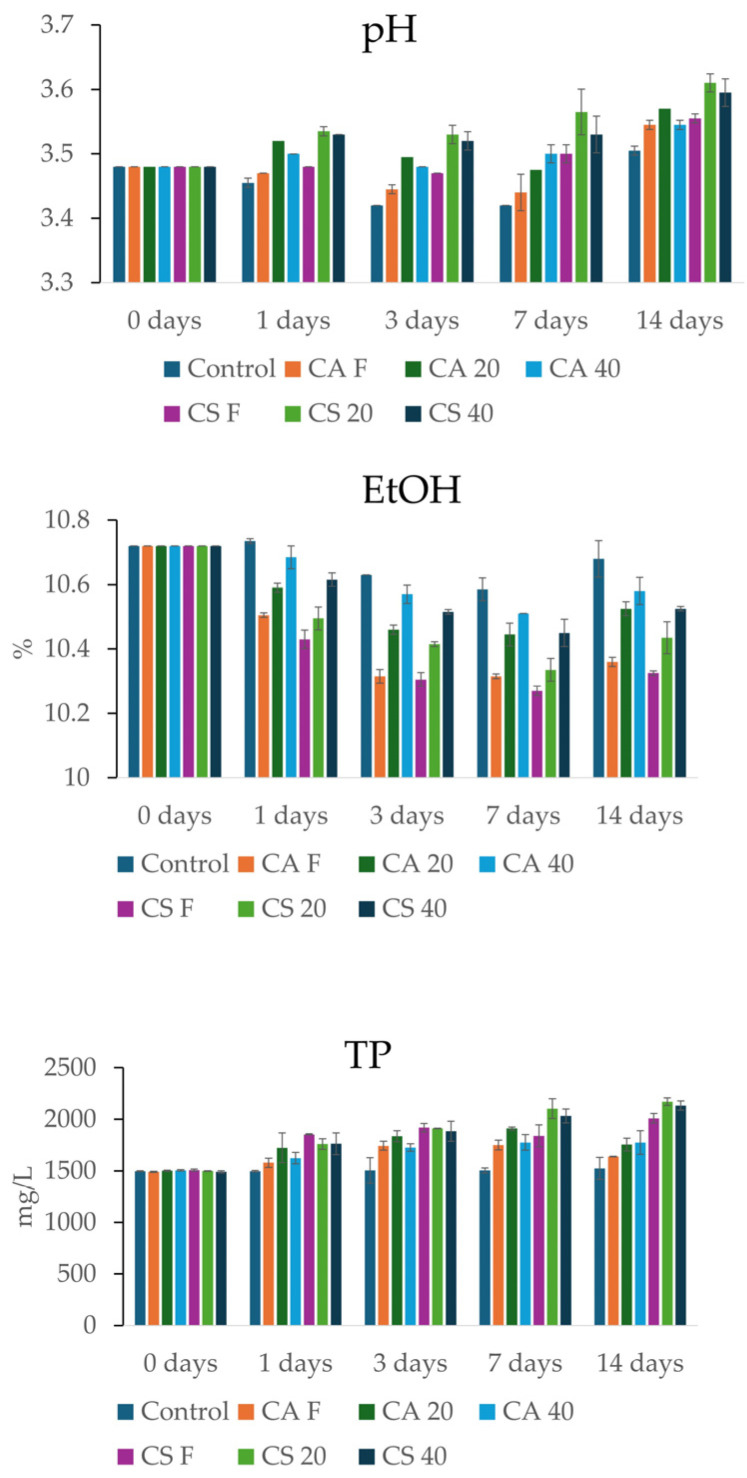
pH, Ethanol (EtOH), and total polyphenols (TP) in the stem samples at 0 and after 1, 3, 7, and 14 days.

**Figure 2:**
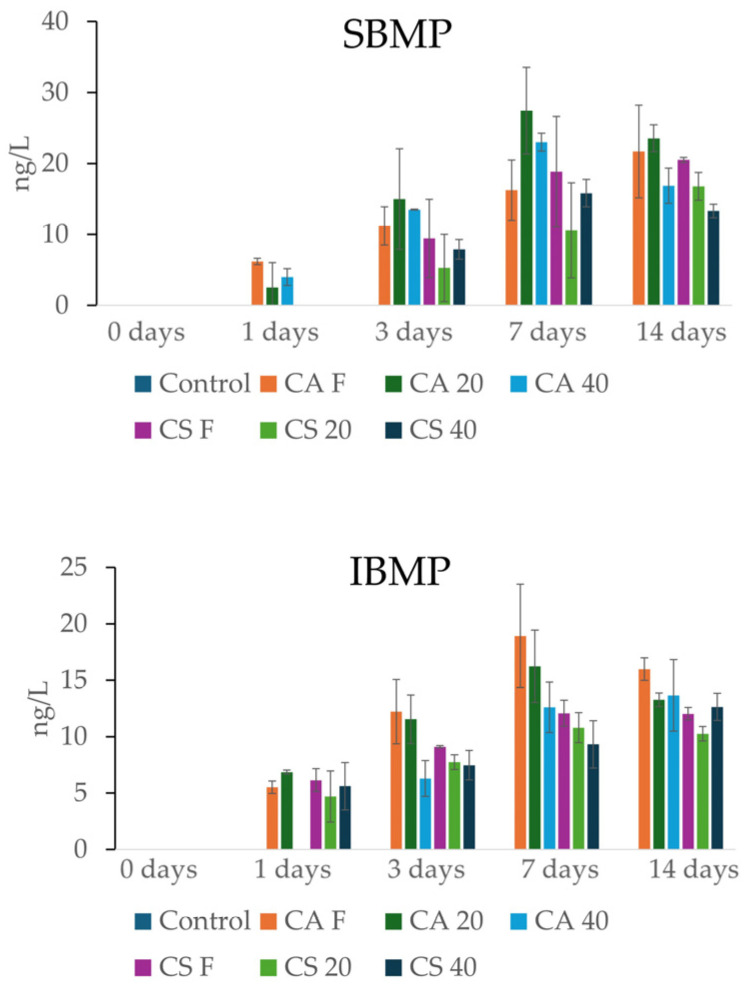
Content of SBMP and IBMP in the stem samples at 0 and after 1, 3, 7, and 14 days.

**Figure 3:**
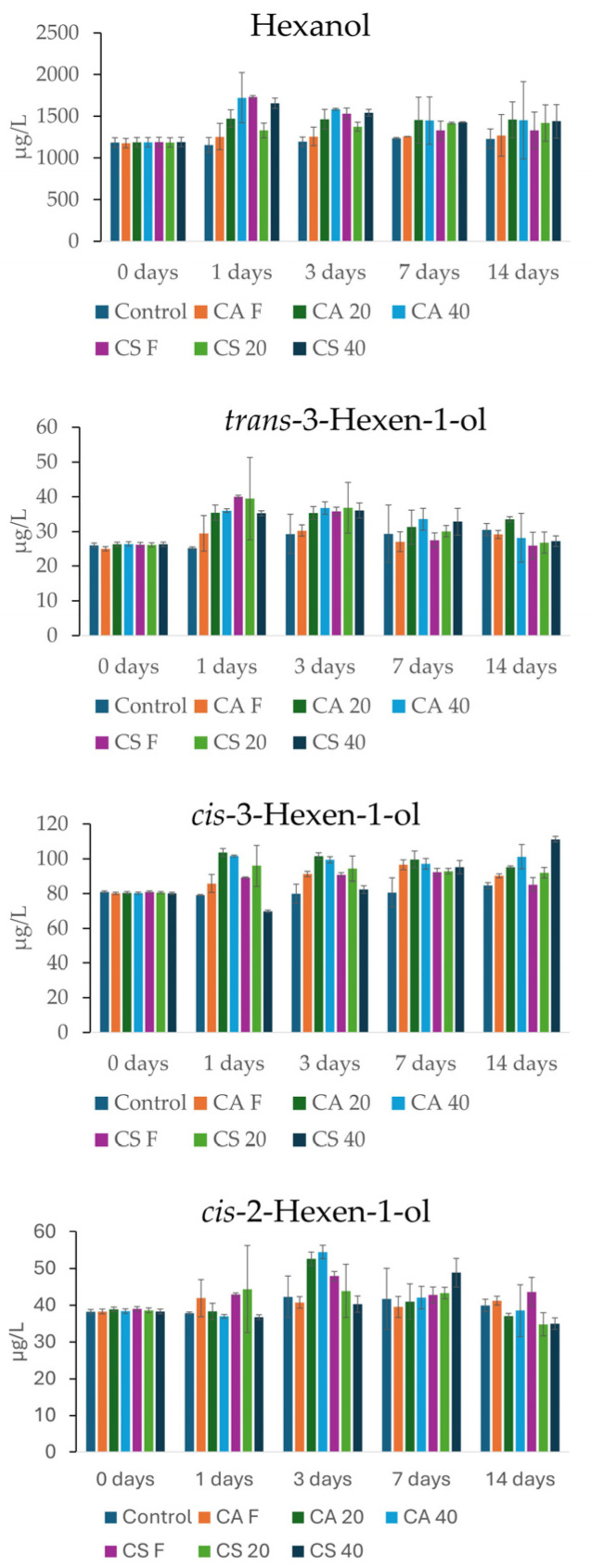
Content of C_6_ alcohols (µg/L) in the stem samples at 0 and after 1, 3, 7, and 14 days.

**Figure 4:**
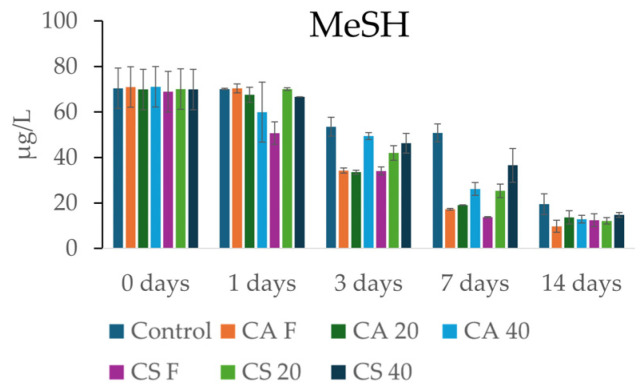
Content of MeSH (µg/L) in the stem samples at 0 and after 1, 3, 7, and 14 days.

The results obtained are shown in Figure 1, Figure 2, Figure 3 and Figure 4, while the ANOVA analysis is reported in Appendix A.1. For the visualization of the entire data set at 14 days, a heat map has been generated and reported in Supplementary Figure S1. Results highlighted a slight but progressive increase in pH across all treatments involving stem addition, compared to the control, which remained substantially stable at a final value of approximately 3.5 (Figure 1). Consistent with previous studies [4,19,20], the observed pH fluctuations were likely driven by changes in the organic acid profile, notably tartaric acid. This phenomenon might be triggered by the leaching of potassium and calcium ions from the grape stems, which promotes the formation and subsequent precipitation of tartrate salts. Regarding the different varieties, withered Cabernet Sauvignon exerted a slightly higher alkalinizing effect than Corvina; specifically, the CS 20 treatment reached a maximum pH value of 3.61 by day 14, showing an increase of 0.1 compared to the control. Concerning the impact of the dehydration process, the data suggested that samples with an intermediate dehydration level achieved higher pH values, whereas the final pH increase in Corvina stems withered at 40% was slightly lower. From a kinetic perspective, the rise in pH was not immediate but required prolonged contact time, with the most significant shifts occurring between the 7th and 14th days of infusion. The pH plays a role in sensory perception: an excessive increase in pH can turn fresh, crisp wines into flat wines and significantly increase bitterness perception [44,45]. Moreover, pH modulates color and aromatic evolution, particularly affecting compounds such as esters and terpenes [46,47].

Regarding ethanol concentration (Figure 1), a decrease was observed across all treatments involving grape stems, whereas the control remained stable. The most pronounced decrease was observed in the samples treated with fresh stems, both Corvina and Cabernet Sauvignon, reaching 10.4% and 10.3%, respectively, by the fourteenth day. Compared to the control, a decrease of 0.32% and 0.35% was observed. A clear correlation with the moisture content of the stems emerged: fresh stems induced the greatest ethanol reduction, followed by those with 20% weight loss, and finally by the samples with 40% weight loss. While previous studies suggest that a decrease in alcohol levels could be attributed to a dilution effect [4], the observed ethanol reduction in withered samples, despite their low water content, suggests that additional mechanisms might be involved. Beyond dilution, it has been reported that the stem surface can capture ethanol molecules [20]. This reduction in ethanol content is a particularly significant finding in the current context of global warming, where rising alcohol levels in wine due to accelerated grape sugar accumulation [48] contrast with the growing consumer preference for lighter-bodied wines [49,50]. Interestingly, the reduction in alcohol content was found to be a rapid phenomenon, with most of the decrease occurring within the first 24 h of contact. In the fresh stem treatments, levels dropped immediately from the initial 10.72% to near 10.40%, before stabilizing around 10.30% by the third day. Conversely, the kinetics for the withered stems (20% and 40% weight loss) exhibited a significantly less steep decline.

The addition of grape stems led to an increase in phenolic concentration within the first 24 h (Figure 1). During this initial phase, the stems released the most soluble and readily available compounds on their surfaces. Subsequently, between days 1 and 7, a progressive extraction phase occurred, driven by the diffusion of tannins and flavonols. Interestingly, CS samples showed higher release compared to CA samples, reaching peaks near 2170 mg/L in the withered treatments; this confirmed either a higher phenolic content or a stem structure that facilitated release.

The dehydration process also played a crucial role in kinetics: stems withered at 20% and 40% showed higher final phenolic content than fresh stems. Compared to the control, CA F and CA 20 samples showed an increase of 10% and 9%, respectively, while CA 40 showed an increase of 20%. In CS, the CS F and CS 20 samples increased by 30% and 33%, respectively, while CS 40 increased by 34%.

This behavior suggested that technical dehydration in the *fruttaio* not only concentrated compounds within the stem tissue but also modified their releasing ability, allowing for a more consistent enrichment of the wine. However, a slight fluctuation or decrease was observed between days 7 and 14 in some treatments (e.g., CA F and CA 20). The slight decrease in phenolic content observed between days 7 and 14 in some treatments might be explained by re-adsorption of polyphenols onto stem cell surfaces or by precipitation following interactions with other wine macromolecules. Previous studies have documented an increase in total phenolic content when stems are present during fermentation [1]. Current evidence suggests that the extent of phenolic enrichment depends on two key factors: varietal characteristics and maceration duration [4,7,12,17,18,20]. In addition to these, drying treatments also appeared to play a significant role [36].

Methoxypyrazines are nitrogenated heterocycles responsible for distinctive herbaceous, green, or even earthy aromas, including green pepper and asparagus [51], typical of wine produced with Cabernet Sauvignon, Sauvignon Blanc, Cabernet Franc, and Merlot noir and other varieties [52,53,54,55,56]. These compounds possess extremely low odor thresholds, typically in the ng/L range. While their origin is primarily varietal, contributing characteristic bell pepper notes to varieties such as Cabernet Sauvignon, Sauvignon Blanc, Cabernet Franc, and Merlot [57], their contribution is not universally considered positive [51]. The analysis of methoxypyrazine release, IBMP, and SBMP underscores that grape stems serve as a significant source of these compounds, with extraction kinetics that develop progressively over the 14-day contact period.

Regarding SBMP, extraction was negligible during the first 24 h, followed by a marked increase starting from the third day onward (Figure 2). The treatments involving CA samples, particularly CA F and CA 20, showed higher release, reaching levels exceeding 20 ng/L, whereas CA 40 showed significantly lower content 16.9 ng/L showing a decrease of 22% compared to CA F. Similarly, CS F and CS 20 showed higher content at the end of 14 days (20.5 ng/L and 16.8 ng/L) than CS 40, showing 13.3 ng/L with a net decrease of 33%. No pyrazines were found in the control.

The kinetics of IBMP followed a similar profile, with extraction becoming significant between the first and third days (Figure 2). Again, CA F showed higher release, stabilizing at approximately 16–18 ng/L between the seventh and fourteenth days. Conversely, Cabernet Sauvignon showed an IBMP release that tended to stabilize or decrease after the third day, with final values of approximately 10–12 ng/L. A fundamental aspect emerging from the data is the impact of stem withering treatments. Withering appears to modulate the release of these herbaceous compounds; specifically, CA 20 and 40 showed a reduction of 17% and 14% of the final SBMP concentration compared to the fresh treatment, suggesting that stem dehydration may limit pyrazine leaching or promote partial degradation. The observed reduction in pyrazine release from withered stems could be explained by at least two non-exclusive mechanisms. First, prolonged dehydration in the fruttaio exposes stem tissues to oxygen, potentially leading to oxidative degradation. Second, withering may induce structural modifications in the stem cell wall, leading to increased exposure to lignin, which might enhance physical adsorption onto the solid matrix, thereby reducing pyrazine extractability into the wine. However, further studies are needed to confirm these hypotheses.

C_6_ compounds are characterized by green and grassy sensory notes. In wine, they are formed from enzymatic oxidation of fatty acids, α-linolenic and α-linoleic, during grape crushing in the pre-fermentative stage [58,59]. The impact of the different treatments on C_6_ compounds was minor (Figure 3). Hexanol showed a slight increase, particularly in the CA 40 and CS 40 treatments; however, the differences were not significant compared to the other treatments. For *trans*-3-hexen-1-ol, all treatments showed a minor but significant increase compared to the control, after which concentrations returned to levels similar to those of the control at 7 and 14 days. *cis*-3-Hexen-1-ol also demonstrated a moderate but significant increase in the treatments containing stems. In this case, an effect of the withering treatment was observable, with withered stems releasing a higher quantity of the compound, in the order 40, 20, and F. Finally, *cis*-2-hexen-1-ol showed a slight increase after 3 days in the withered CA treatments. However, no significant differences were observed between 7 and 14 days.

Methanethiol, along with other volatile sulfur compounds such as hydrogen sulfide and ethanethiol, is frequently synthesized during winemaking or during storage. It is primarily formed via the degradation of sulfur-containing amino acids (especially methionine) by yeast during alcoholic fermentation, as well as through the chemical or microbial breakdown of cysteine and glutathione. These compounds adversely affect wine aroma, overall perceived quality, and consumer acceptance due to their unpleasant odors, often described as reminiscent of cabbage, onion, and garlic [60,61,62,63,64].

The kinetic analysis of MeSH revealed a consistent decrease across all treatments throughout the 14-day experimental period (Figure 4). Significant effects of both grape variety and stem treatment were observed as early as the first 24 h. Notably, CS F induced the most immediate reduction, with MeSH levels dropping to 50.7 µg/L, whereas the control remained stable above 70 µg/L. On day 1, CS F was the only treatment to show a significant decrease in MeSH compared to the control, achieving a reduction of approximately 18 µg/L. The most intensive decrease phase occurred between the third and seventh days for all samples, including the control, resulting in concentrations significantly lower than both the initial and control values. By day 14, the lowest methanethiol concentrations were recorded across all experimental groups. While the gap between the control and the treatments narrowed toward the end of the trial, the differences remained statistically significant; CA F achieved the lowest absolute residual concentration at 9.73 µg/L.

The extent of this final decrease, with nearly all stem samples settling between 9 and 14 µg/L compared to 19.45 µg/L in the control, confirms that the grape stems act as an active sequestrant for sulfur-based off-odors. Compared to the control, MeSH reductions ranged from 24% for CS 40 to 50% for CA F. The fact that the control during the 14 days showed a lower but significant decrease compared to stem samples was most likely due to competition with other wine matrix compounds and with other molecules for binding to VSCs. This suggests that stem addition could serve as a natural and sustainable strategy for mitigating reduction defects in wine. Such a decrease in reductive thiol compounds is attributable to the formation of bonds between sulfhydryl groups and tannins [38], a class of polyphenols particularly abundant in grape stems [34].

In summary, the addition of grape stems to the wine matrix leads to a pyrazine enrichment that is strictly dependent on both the variety and the degree of material withering. These findings are crucial for winemakers, as they indicate that the use of withered stems can be a strategic approach to capitalize on the abatement of reductive thiol, such as MeSH, while simultaneously minimizing the input of undesirable vegetal off-odors.

### 3.2. Evaluation of the Adsorption Capacity of Grape Stems

A key question concerns whether the reduction in MeSH is mediated by adsorption onto the grape stem itself or by the reactivity of tannins released into the solution. The experimental data (Table 3) allowed the differentiation between the effect of the solid matrix and the soluble components leached into the wine. The control sample had the highest MeSH concentration (37.61 µg/L), whereas the SI treatment showed the lowest (6.4 µg/L). This treatment proved to be the most effective, achieving a reduction of over 80% compared to the control. In contrast, the SE and ES treatments resulted in concentrations of 21.16 µg/L and 14.68 µg/L, respectively.

The higher methanethiol adsorption capacity of the ES sample compared to the SE sample could be tentatively explained by two factors. First, the solid stem matrix provides a high surface area that may physically adsorb MeSH within hours, whereas soluble leached compounds are present at much lower concentrations and require time to be released into solutions. Second, the condensed tannins released into solution might be smaller and partially oxidized due to radical scavenging activity and could therefore be less reactive toward MeSH than their immobilized counterparts within the stem cell wall. These proposed mechanisms require further investigation.

### 3.3. Evaluation of the MeSH Adsorption Capacity of Commercial Enological Tannins

Given the ability of stems to decrease methanethiol content, the capacity of other enological products, such as commercial tannins, was evaluated. Enological tannins are widely employed during winemaking and aging to clarify and stabilize musts and wines; they function by binding proteins to prevent ferric casse, while also stabilizing color and enhancing both antioxidant activity and sensory properties [65,66,67,68].

**Figure 5:**
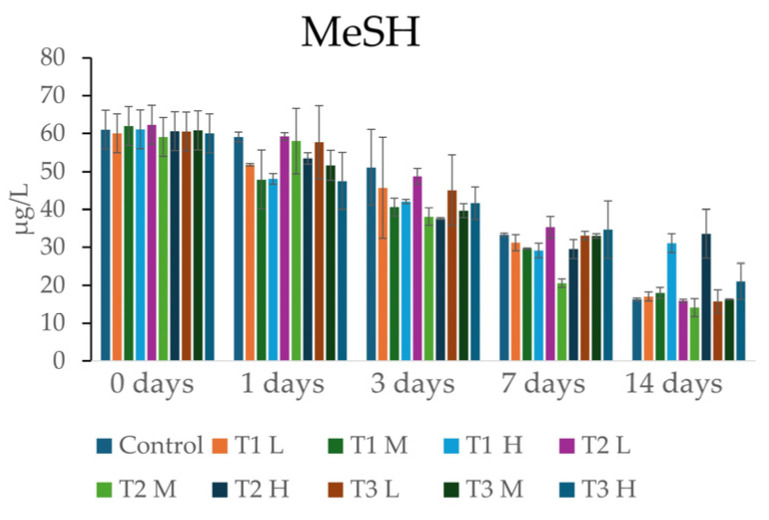
Content of MeSH (µg/L) in the enological tannins’ samples at 0 and after 1, 3, 7, and 14 days.

This type of product, similarly to grape stems, contains tannins [68,69]. These products can form bonds with the sulfhydryl groups of mercaptans. Previously, Bekker et al. (2025) [38] observed a decrease in certain reductive thiols following the addition of enological tannins, although the effect was not always significant depending on the contact time. The experiment was conducted similarly to the stem experiment, where three types of commercial tannins were added at three different concentrations to the same red wine. The maximum concentrations used were those specified by the manufacturer in the technical data sheet. The samples were analyzed at time 0 and after 1, 3, 7, and 14 days; the results are reported in Figure 5, while ANOVA analysis is reported in Appendix A.2.

The results indicated only minor, non-significant differences between treatments during the first seven days of the study. However, by the 14th day, two distinct groups emerged: the first comprised control and all samples treated with low and medium tannin dosages, which showed lower residual concentrations of MeSH, the second comprised all treatments at high concentrations, which retained significantly higher levels of the compound.

These findings contradict previous studies, such as those by Bekker et al. (2025) [38], in which tannins can reduce the content of reductive mercaptans. In our study, conversely, higher tannin concentrations resulted in a less pronounced decrease in MeSH levels. The investigation into the adsorption capacity of enological tannins toward MeSH revealed a counterintuitive phenomenon: as the tannin dosage increases from low to high, the efficacy of the treatment in removing this off-odor does not increase but, in several instances, decreases drastically. T1 H and T2 H exhibited residual MeSH concentrations twice those of the control, reaching 31.08 µg/L and 33.55 µg/L, respectively. Furthermore, the T3 H treatment showed a 1.5-fold higher MeSH content compared to the control.

One hypothesis for this anomalous behavior lies in the intrinsic antioxidant activity of polyphenolic compound [70]. The discrepancy between the present findings and those of Bekker et al. (2025) [38] may be related to the higher tannin concentrations used in this study (500 mg/L vs. approximately 100 mg/L). It can be hypothesized that the introduction of a high tannin dose led to a reduction in the redox potential of the system, interfering with the degradation pathways of methanethiol. Under standard conditions, MeSH can undergo spontaneous oxidation.

However, high content of tannins may act as an oxidative ‘buffer,’ sequestering dissolved oxygen and neutralizing the free radicals that would otherwise facilitate this chemical transformation. It is therefore hypothesized that at such high doses, the antioxidant capacity of tannins may protect MeSH from oxidative degradation, although further studies are required to confirm this mechanism. Consequently, high concentrations of polyphenols might exert an unintended protective effect on the MeSH molecule, preserving it from the natural degradation observed in the control sample. This hypothesis is indirectly supported by Fracassetti et al. (2021), who demonstrated that hydrolysable tannins effectively prevent the formation of MeSH under light exposure [71,72].

## 4. Conclusions

This research evaluated the behavior of grape stems in red wine, aiming to characterize the dynamics of release and adsorption across two varieties, Corvina and Cabernet Sauvignon, and different withering degrees.

Analysis of enological parameters revealed that the addition of stems leads to a pH increase, which was more pronounced in withered samples. Furthermore, a reduction in alcohol content was observed in stem-treated samples. Although higher in fresh stem samples, the ethanol decrease was also observed for withered stems, suggesting that the mechanism is not a simple dilution effect but likely involves physical interaction between the stem surface and ethanol molecules. Regarding phenolic compounds, stem-treated samples showed an increase compared to the control, influenced by both variety and withering treatment. Cabernet Sauvignon showed higher release levels than Corvina; moreover, withered stems led to a higher final phenolic enrichment compared to fresh ones. Concerning volatile compounds responsible for herbaceous notes, the study highlighted stems as a source of methoxypyrazines. However, the use of withered stems proved to be an effective treatment to limit the release of these compounds. Concerning methanethiol, kinetic trends highlighted the ability of all stem types to decrease MeSH content. The stems acted as an active substrate in sequestering undesirable sulfur compounds, primarily via direct adsorption onto their solid surface. Comparison with commercial enological tannins showed an unexpected trend. While no significant differences were observed initially, by day 14, the highest tannin dosages led to significantly higher residual methanethiol concentrations compared to the control and lower dosages. These results, seemingly contradictory to the existing literature, may be explained by the high antioxidant activity of tannins at high doses. By acting as oxygen and free-radical scavengers, they may inhibit the oxidative degradation of methanethiol to dimethyl disulfide, thereby preserving the thiol in its original form. Limitation of the study: the experimental design, while controlled, necessarily simplifies real winemaking conditions. The use of laboratory-scale macerations cannot fully capture the physicochemical and microbial complexity of industrial fermentations. Additionally, the stem-to-wine ratio used in this study (150 g/L) is higher than the typical stem content of grape clusters, which generally ranges from 2.5% to 7.5% of grape weight. This higher ratio was deliberately adopted to amplify the kinetic signals under controlled laboratory conditions, allowing the reliable detection and quantification of release and adsorption phenomena over a short maceration period.

## Figures and Tables

**Table 1:** Sample list, stem varieties, whitering treatment, and humidity.

Samples	Varieties	Whitering	Humidity
Control	Model wine	Not applicable	Not applicable
CA FR	Corvina	No	76.9%
CA 25	Corvina	20%	52.3%
CA 50	Corvina	40%	27%
CS FR	Cabernet Sauvignon	No	69.3%
CS 25	Cabernet Sauvignon	20%	44.6%
CS 50	Cabernet Sauvignon	40%	19.6%

**Table 2:** Samples, tannins’ origins, and concentration.

Samples	Tannin Origins	Concentration
T1 L	grape	25 mg/L
T1 M	grape	250 mg/L
T1 H	grape	500 mg/L
T2 L	grape seed	25 mg/L
T2 M	grape seed	250 mg/L
T2 H	grape seed	500 mg/L
T3 L	Green tea	25 mg/L
T3 M	Green tea	250 mg/L
T3 H	Green tea	500 mg/L

**Table 3:** Content ± standard deviation of MeSH and significance according to ANOVA.

Samples	Content (µg/L)	Percentage Decrease (%)	S ^1^
Control	37.61 ± 3.52	0	a
SI	6.395 ± 0.64	83	d
SE	21.16 ± 5.07	44	b
ES	14.68 ± 1.26	61	c

^1^ S means significance according to ANOVA (α = 0.05) post hoc Tukey test, lower case letter refers to different groups.

## Data Availability

The original contributions presented in the study are included in the article/Supplementary Material, further inquiries can be directed to the corresponding authors.

## Supplementary Materials

The following supporting information can be downloaded at: https://www.mdpi.com/article/10.3390/foods15101707/s1, Supplementary Table S1. Retention indices, quantification ions, limit of detection (LOD), limit of quantification (LOQ), and repeatability of the analysed compounds. Supplementary Figure S1. Heat map and hierarchical cluster analysis of samples and enological parameters and volatile organic compounds of T14 samples.

## Appendix A

### Appendix A.1. Different Groups According to ANOVA (α = 0.05) Post Hoc Tukey Test for the Stem Experiment

**pH**	**0 d**	**1 d**	**3 d**	**7 d**	**14 d**	**Hexanol**	**0 d**	**1 d**	**3 d**	**7 d**	**14 d**
Control	a	a	a	a	a	Control	a	a	a	a	a
CA F	a	b	b	ab	b	CA F	a	ab	ab	a	a
CA 20	a	e	e	b	c	CA 20	a	c	c	b	a
CA 40	a	d	d	c	b	CA 40	a	cd	c	ab	a
CS F	a	c	c	c	b	CS F	a	d	c	ab	a
CS 20	a	f	f	d	d	CS 20	a	b	bc	b	a
CS 40	a	f	f	cd	d	CS 40	a	d	c	b	a
EtOH	0 d	1 d	3 d	7 d	14 d	trans-3-Hexen-1-ol	0 d	1 d	3 d	7 d	14 d
Control	a	d	e	e	f	Control	a	a	a	a	a
CA F	a	b	b	b	b	CA F	a	ab	a	a	a
CA 20	a	c	c	c	de	CA 20	a	b	b	a	a
CA 40	a	d	d	d	e	CA 40	a	b	b	a	a
CS F	a	a	a	a	a	CS F	a	c	b	a	a
CS 20	a	b	ba	b	c	CS 20	a	bc	ab	a	a
CS 40	a	c	c	c	d	CS 40	a	b	b	a	a
TP	0 d	1 d	3 d	7 d	14 d	cis-3-Hexen-1-ol	0 d	1 d	3 d	7 d	14 d
Control	a	a	a	a	a	Control	a	b	a	a	a
CA F	a	b	bc	b	ab	CA F	a	c	b	b	b
CA 20	a	c	c	c	b	CA 20	a	d	c	b	c
CA 40	a	bc	b	b	b	CA 40	a	d	c	b	d
CS F	a	c	c	bc	c	CS F	a	c	b	b	a
CS 20	a	c	c	d	d	CS 20	a	cd	bc	b	b
CS 40	a	c	c	d	d	CS 40	a	a	a	b	e
SBMP	0 d	1 d	3 d	7 d	14 d	cis-2-Hexen-1-ol	0 d	1 d	3 d	7 d	14 d
Control	-	-	-	-	-	Control	a	a	a	a	a
CA F	-	a	c	c	c	CA F	a	ab	a	a	a
CA 20	-	a	c	bc	b	CA 20	a	a	c	a	a
CA 40	-	-	a	ab	bc	CA 40	a	a	c	a	a
CS F	-	a	b	ab	b	CS F	a	b	b	a	a
CS 20	-	a	a	ab	a	CS 20	a	ab	ab	a	a
CS 40	-	a	a	a	b	CS 40	a	a	a	a	a
IBMP	0 d	1 d	3 d	7 d	14 d	MeSH	0 d	1 d	3 d	7 d	14 d
Control	-	-	-	-	-	Control	a	b	d	d	d
CA F	-	a	ab	ab	bcd	CA F	a	b	a	a	a
CA 20	-	a	ab	b	d	CA 20	a	b	a	a	b
CA 40	-	a	b	b	b	CA 40	a	ab	d	b	b
CS F	-	-	ab	ab	c	CS F	a	a	a	a	b
CS 20	-	-	a	a	b	CS 20	a	b	b	b	b
CS 40	-	-	a	ab	a	CS 40	a	b	bc	c	b
Lowercase letters indicate significantly different groups according to one-way ANOVA (α = 0.05) followed by Tukey’s post hoc test.											

### Appendix A.2. Different Groups According to ANOVA (α = 0.05) Post Hoc Tukey Test for the Enological Tannins Experiment

**MeSH**	**0 d**	**1 d**	**3 d**	**7 d**	**14 d**
Control	a	b	a	b	a
T1 L	a	a	a	b	a
T1 M	a	a	a	b	a
T1 H	a	a	a	b	b
T2 L	a	ab	a	b	a
T2 M	a	ab	a	a	a
T2 H	a	a	a	b	b
T3 L	a	ab	a	b	a
T3 M	a	a	a	b	a
T3 H	a	a	a	b	a
Lowercase letters indicate significantly different groups according to one-way ANOVA (α = 0.05) followed by Tukey’s post hoc test.					
