# Supplementary material for: From Waste to Taste: Dynamic Interaction of Grape Stems with Wine Off-Odors
Source: Foods. 2026 May 13;15(10):1707. doi: 10.3390/foods15101707 (PMC13206134; doi:10.3390/foods15101707)
Supplement: Supplementary file 1 [file foods-15-01707-s001.zip › foods-4278986-supplementary.pdf]

Supplementary Table S1. Retention indices, quantification ions, limit of detection (LOD), limit of quantification (LOQ), and repeatability of the analysed compounds.

	EM1	LRI2	Identification3	Quantitation ion m/z	Qualifier ions m/z	LOD (µg/L)	LOQ (µg/L)	RSD%4
Methanthiol	a	645	RS	47	48	0.09	0.27	8.8
IBMP		1181	RS	124	151, 166	0.8 (ng/L)	0.24 (ng/L)	3.2
SBMP		1081	RS	124	138, 151	0.6 (ng/L)	0.2 (ng/L)	4.1
Hexanol		1359	RS	56	69, 84	3.4	1.2	1.2
trans-3-Hexen-1-ol		1388	RS	67*	82, 55	2.3	0.8	1.7
cis-3-Hexen-1-ol		1394	RS	67	82, 55	4.2	1.4	1.9
cis-2-Hexen-1-ol		1416	RS	67	82, 57	3.3	1.1	0.8

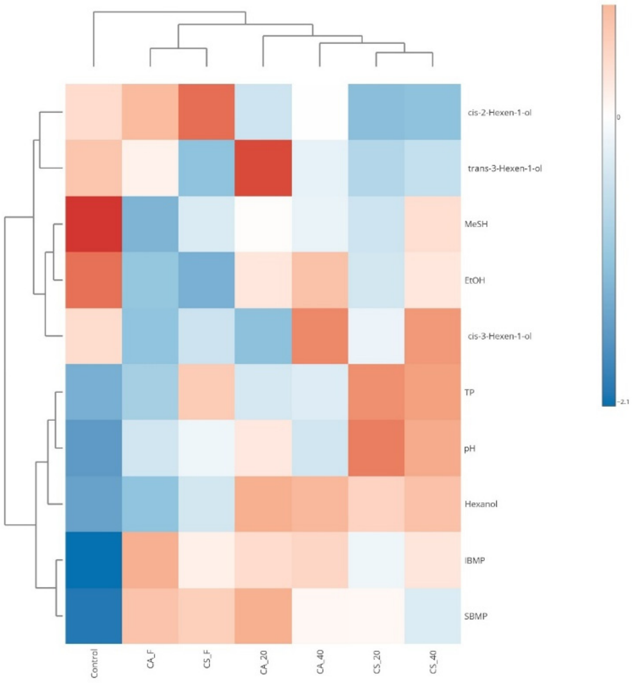

Supplementary Figure S1 Heat map and hierarchical cluster analysis of samples and enological parameters and volatile organic compounds of T14 samples.
